# Supplementary material for: The activity of TRAF RING homo- and heterodimers is regulated by zinc finger 1
Source: Nat Commun. 2017 Nov 27;8:1788. doi: 10.1038/s41467-017-01665-3 (PMC5702613; doi:10.1038/s41467-017-01665-3)
Supplement: Supplementary file 1 — Supplementary Information [file 41467_2017_1665_MOESM1_ESM.pdf]

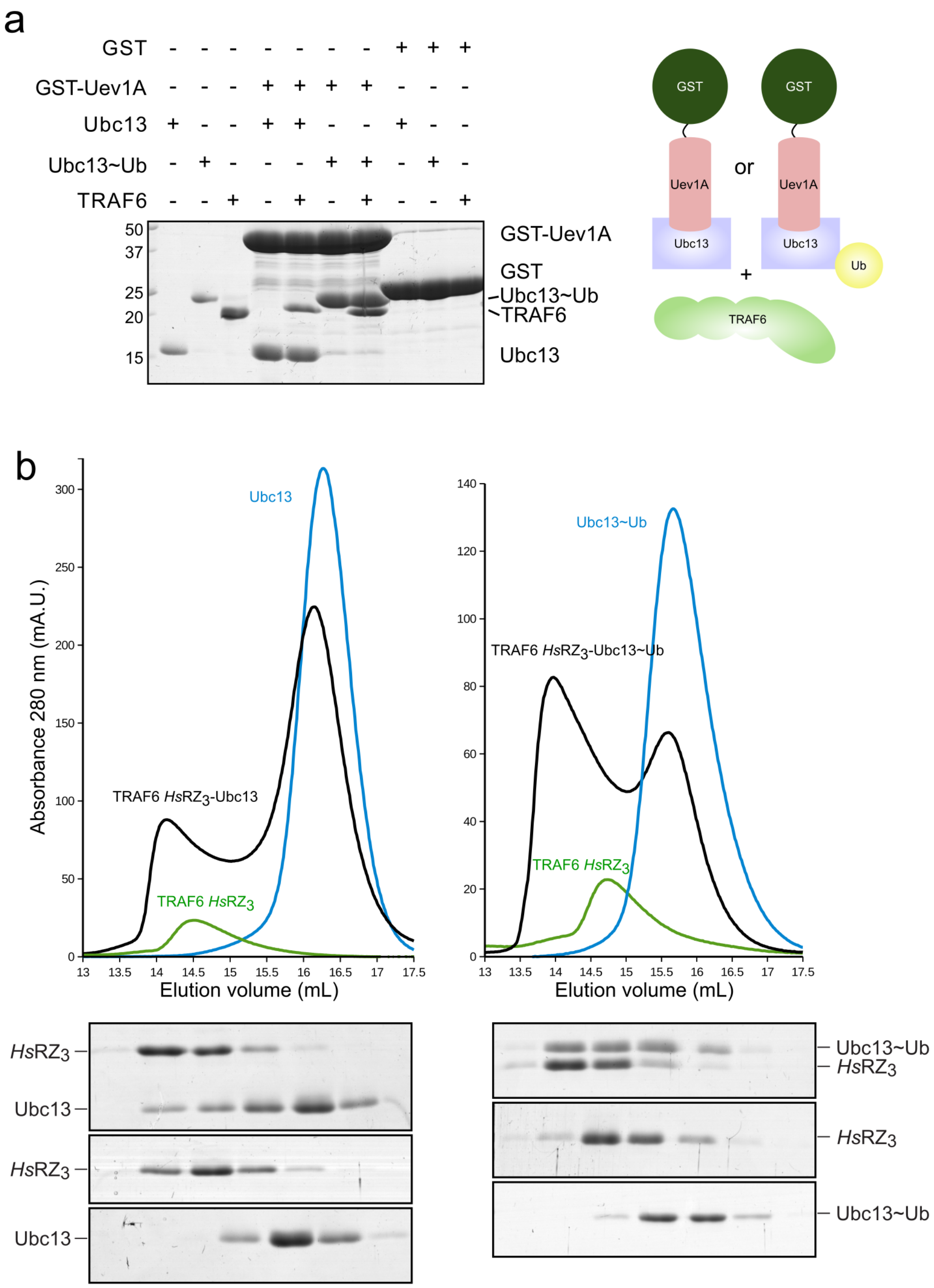

Supplementary Figure 1: TRAF6 preferentially binds the Ubc13~Ub conjugate. Related to Figure 1. **(a)** GST pull-down of GST-fused Uev1A mixed with either Ubc13 or Ubc13~Ub, and *Hs*RZ$_3$. **(b)** Analytical size-exclusion chromatography of 50 µM *Hs*RZ$_3$, Ubc13 (left), and Ubc13~Ub conjugate (right) with identical fractions shown below.

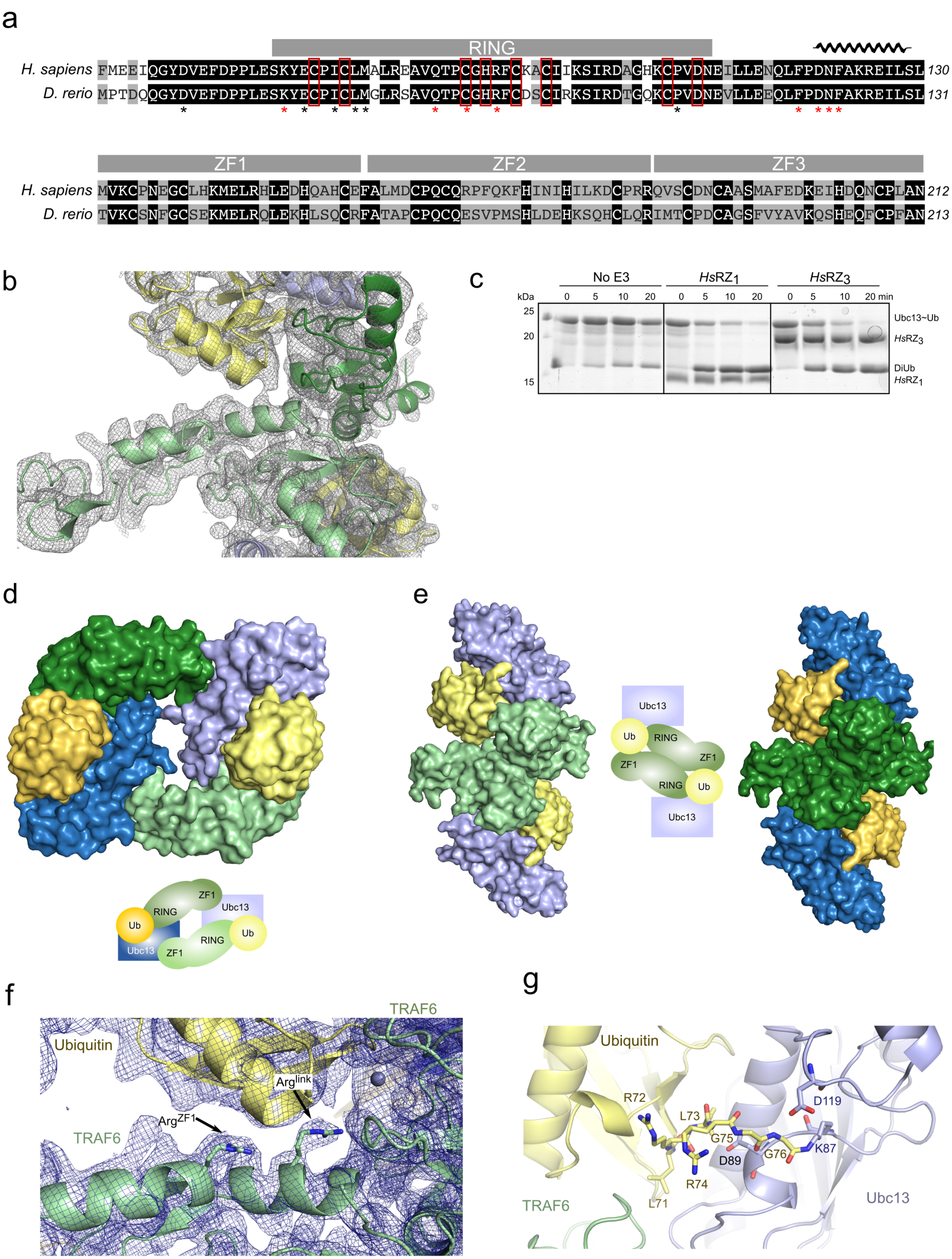
a
RING
H. sapiens FMEEIQGYDVEFDPPLESKYECPICLMALREAVQTPCGHRFCKACIIKSIRDAGHKCPVDNEILLENQLFPDNFAKREILSL 130
D. rerio MPTDQQGYDVEFDPPLESKYECPICLMGLRSAVQTPCGHRFCDSCIRKSIRDTGQKCPVDNEVLLEEQLFPDNFAKREILSL 131
ZF1
ZF2
ZF3
H. sapiens MVKCPNEGCLHKMELRHLEDHQAHCEFALMDCPQCQRPFQKFHINIHILKDCPRRQVSCDNCAASMAFEDKEIHDQNCPLAN 212
D. rerio TVKCSNFGCSEKMELRQLEKHLSQCRFATAPCPQCQESVPMSHLDEHKSQHCLQRIMTCPDCAGSFVYAVKQSHEQFCPFAN 213
b
c
No E3
HsRZ1
HsRZ3
kDa
0 5 10 20 0 5 10 20 0 5 10 20 min
25
20
15
Ubc13~Ub
HsRZ3
DiUb
HsRZ1
d
RING
ZF1
Ubc13
Ub
Ubc13
ZF1
RING
Ub
e
Ubc13
Ub
RING
ZF1
ZF1
RING
Ub
Ubc13
f
TRAF6
Ubiquitin
Arglink
ArgZF1
TRAF6
g
Ubiquitin
R72
L73
G75
D119
G76
K87
D89
R74
L71
TRAF6
Ubc13

Supplementary Figure 2: Structural characterisation of TRAF6 in complex with a Ubc13~Ub conjugate. Related to Fig. 1 and 2. **(a)** Sequence alignment of TRAF6 from *H. sapiens* and *D. rerio* with identical residues highlighted in black, and similarity shown in grey. Zinc-coordinating residues are boxed in red, and residues at the dimerisation and E2 interfaces are indicated with red and black asterisks, respectively. (**b**) A 2Fo-Fc composite omit map of *Dr*RZ_3 contoured at 1σ level (shown in grey). Protein ribbons are coloured as in Fig. 1d. **(c)** Coomassie-stained single-turnover discharge assay comparing the activity of *Hs*RZ_1 and *Hs*RZ_3. **(d)** Surface representation of the asymmetric unit of the *Dr*RZ_1-Ubc13~Ub structure. The asymmetric unit contains two complexes that each form half of the dimeric biological unit. **(e)** Dimeric complexes generated by crystallographic symmetry mates for the RZ_1 complex. **(f)** A 2Fo-Fc electron density map (contoured at 1.1σ level) showing the density for the Arg residues critical for ubiquitin transfer. Arg126 and Arg147 are labelled as Arg^{link} and Arg^{ZF1} for clarity. **(g)** Details of the interaction between the C-terminal tail of ubiquitin and Ubc13. Coloured as in panel **e**).

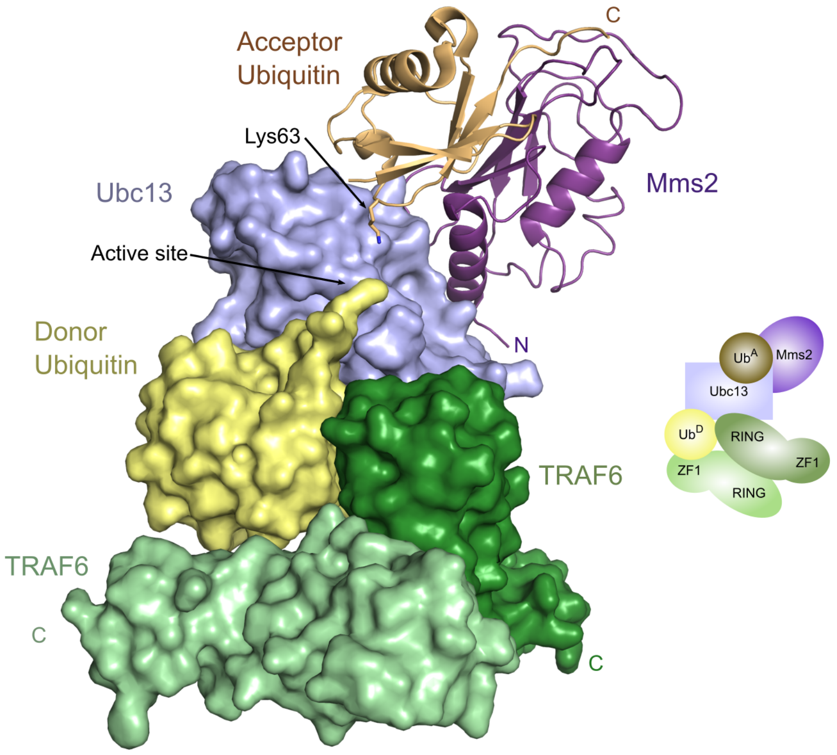

Supplementary Figure 3: Model of the Ubc13 catalytic complex is compatible with the TRAF6 crystal structure. Ubc13 from the Ubc13~Ub/Mms2 complex (PDB ID: 2GMI)[1] was overlayed onto Ubc13 from the TRAF6 complex. Mms2 and ubiquitin are shown in ribbon representation, while Lys63 of the acceptor ubiquitin (Ub^A) is shown as sticks close to the C-terminal tail of the donor ubiquitin (Ub^D) (nitrogen atom coloured blue).

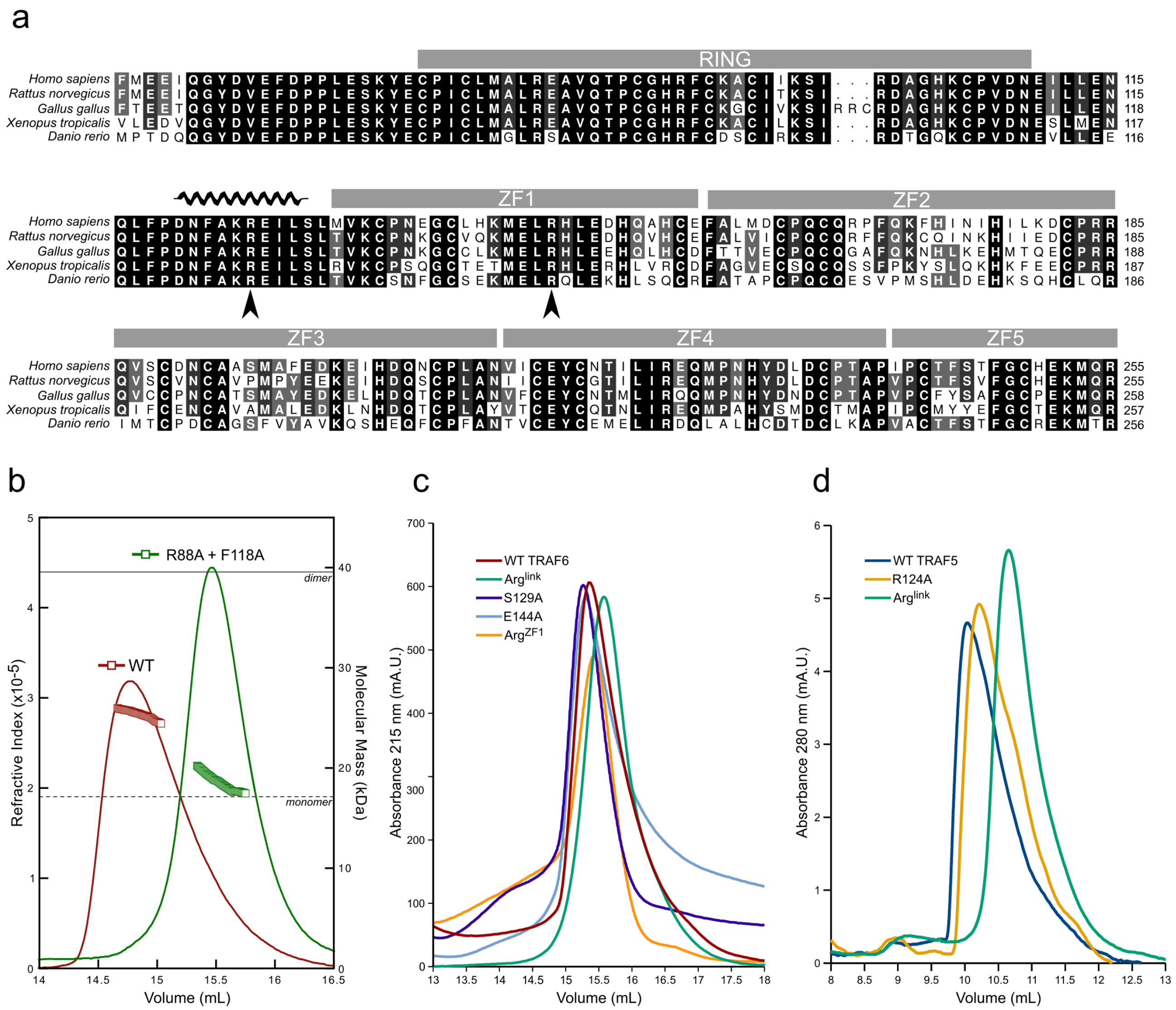

Supplementary Figure 4: Analysis of mutations from the RING and ZF of TRAF6. (**a**) Alignment of the RING and ZFs of TRAF6. The conserved Arg^{link} and Arg^{ZF1} are indicated with arrows. (**b**) Multi-angle light scattering analysis for WT TRAF6 (red) and a mutant TRAF6 in which the dimer interface is disrupted[2] (green). For both runs, proteins were at 50 μM. Elution profile shown as lines, while squares represent calculated molecular mass. (**c**) Overlay of size-exclusion chromatograms showing the elution profile of TRAF6 mutant proteins analysed in Fig 2. Chromatography runs performed at 40 μM. (**d**) Overlay of size-exclusion chromatograms of wild-type TRAF5 and mutants performed at 40 μM.

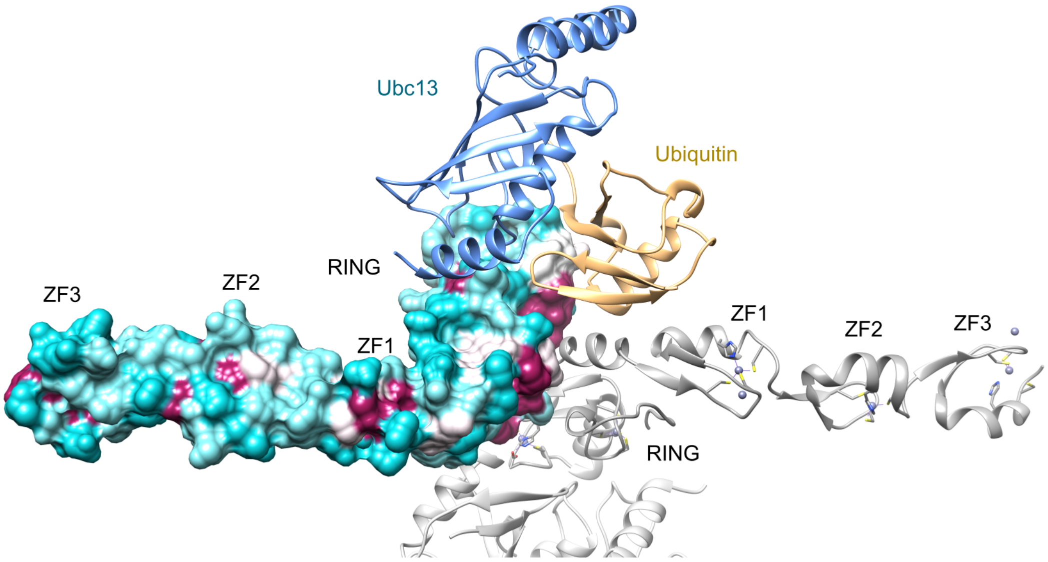

Supplementary Figure 5: The E2 interface is not conserved between TRAF2, 3, 5, and 6.

Conservation surface mapping identical to that in **Fig. 4a**, but oriented to show the E2-binding interface on TRAF6. Ubc13 (blue) and ubiquitin (beige) are shown as ribbons and are modelled onto *Hs*RZ_3 using the crystal structure presented in the main text.

Figure 1c

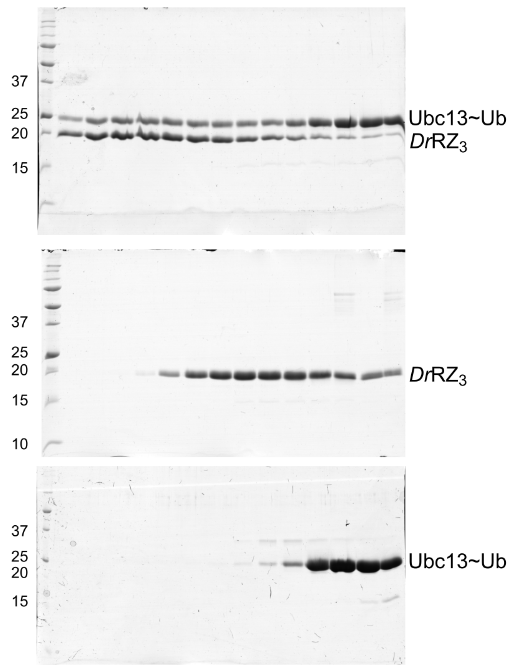

Fig. 2c

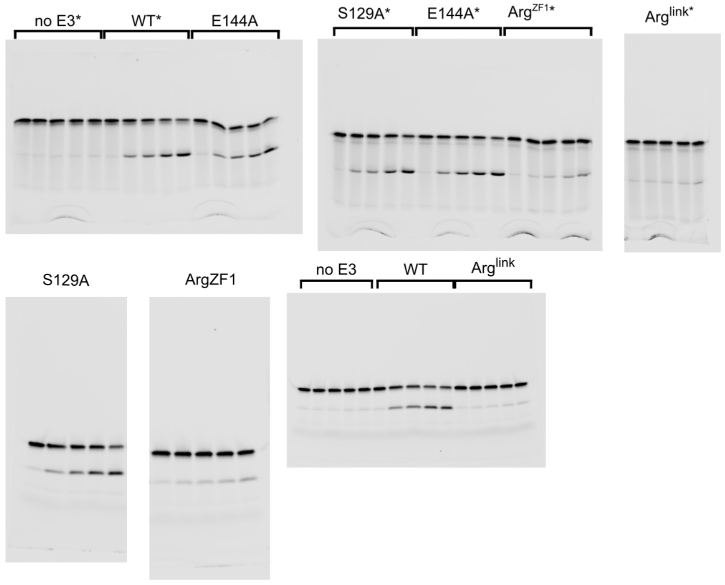

Supplementary Figure 6. Uncropped gels for Fig. 1 & 2. Gels shown in Fig 2c are indicated with an asterisk. Also shown are the gels from the duplicate experiment for Fig 2d.

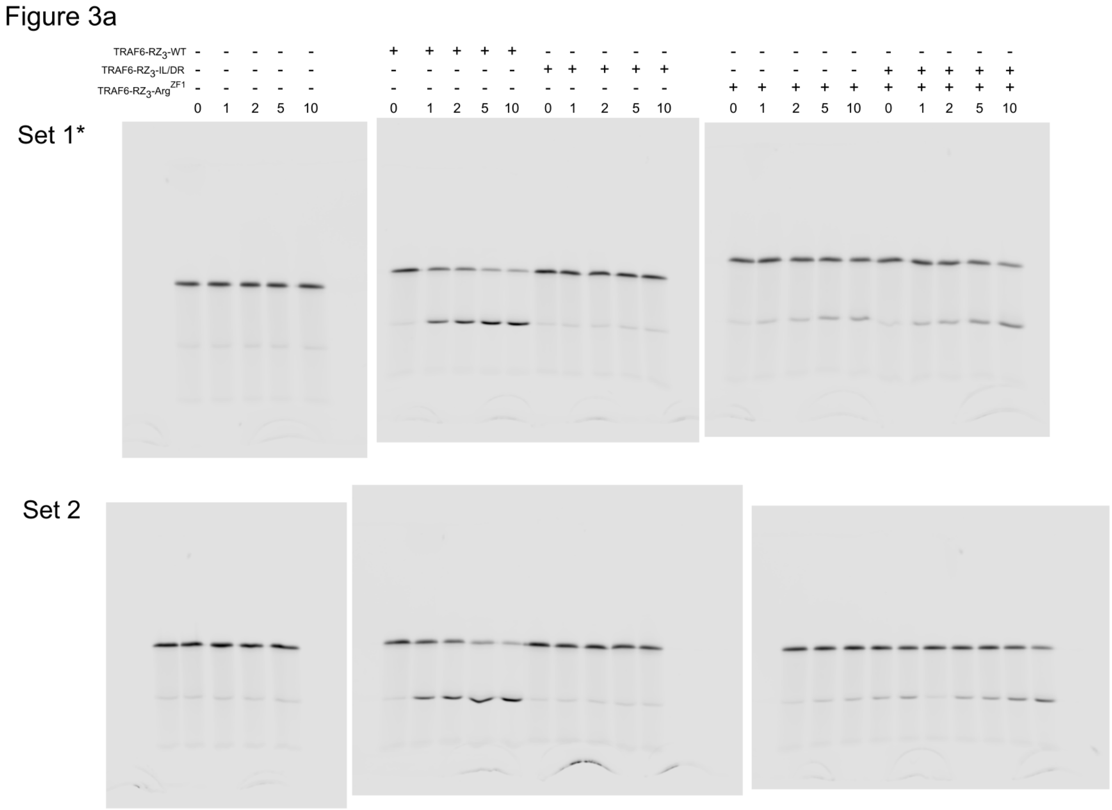

Supplementary Figure 7. Uncropped gels for Fig 3. Gels shown in Fig 3a are indicated with an asterisk (Set 1*). Also included are the gels from the duplicate experiment (Set 2) for Fig 3b.

Figure 4c

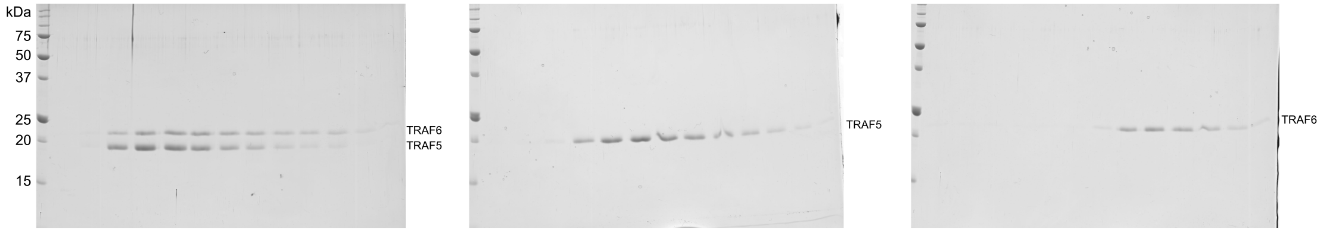

Figure 4d

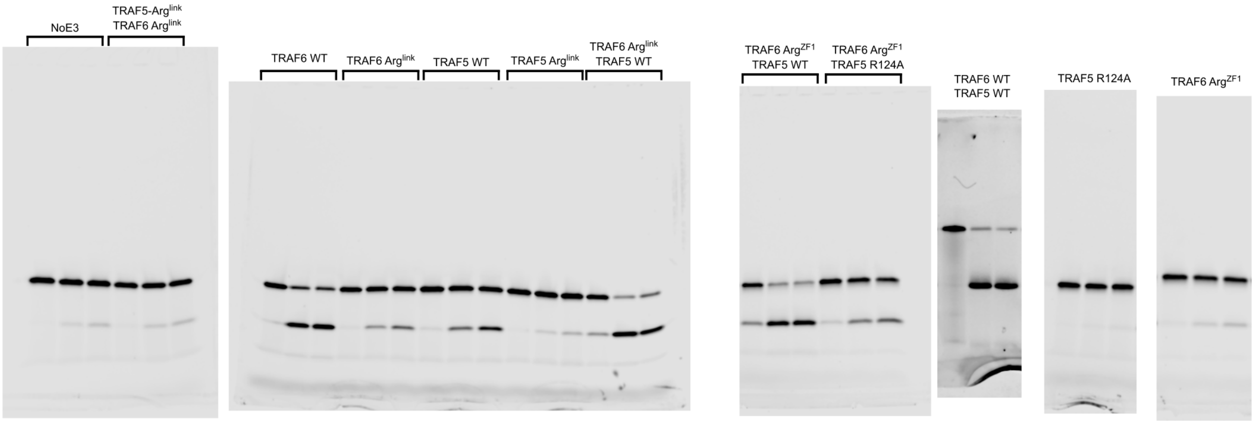

Supplementary Figure 8. Uncropped gels for Fig. 4.

Supplementary 1a

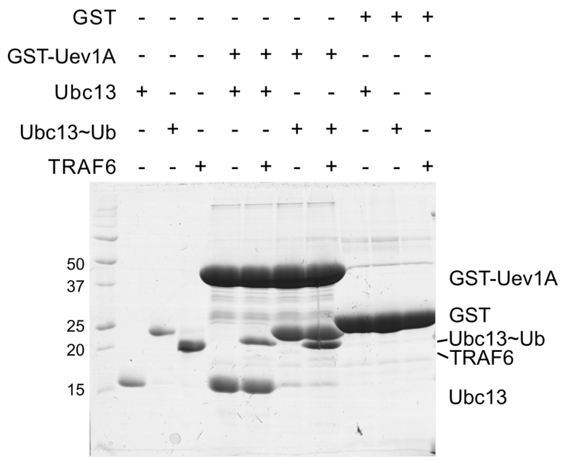

Supplementary 1b

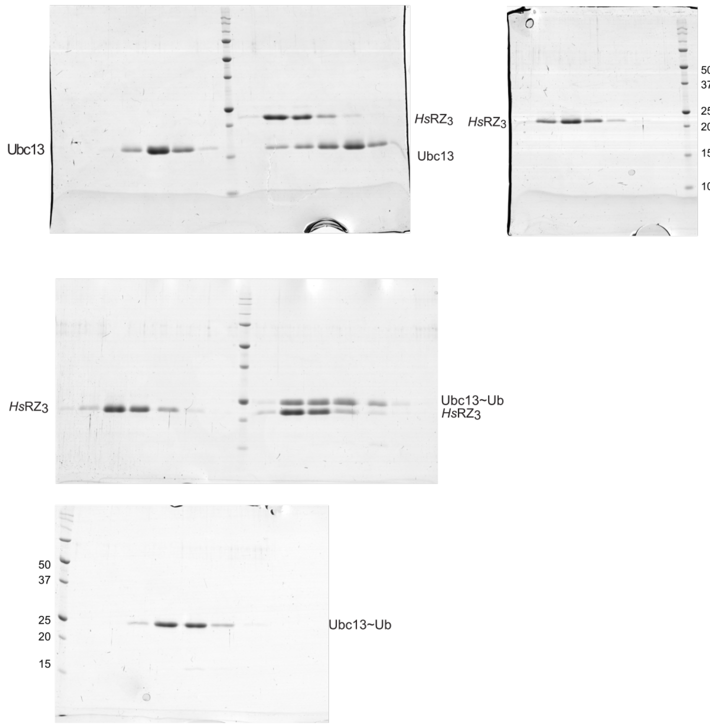

Supplementary 2c

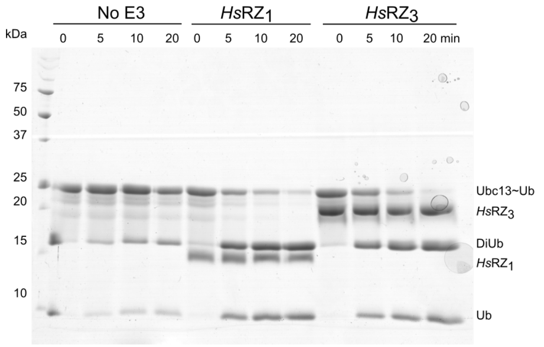

Supplementary Figure 9. Uncropped gels for Supplementary Information.

Supplementary Table 1. List of primers.

Primer Name	Sequence (5' - 3')
*Hs*TRAF6_Forward	GCGCCATGGAGGAGATCCAGGGATATGATGTAGAG
*Hs*TRAF6_RZ3_Reverse	GCGCTCGAGTGCCAAAGGACAGTTCTGGTCATGGATCTCTTTATC
*Hs*TRAF6_RZ1_Reverse	GCGCTCGAGAAGAGCAAACTCACAATGTGCTTGATGATCCTC
*Hs*TRAF6_R125A_Forward	AAAGCTGAGATTCTTTCTCTGATGGTGAAATGTCCAAATGAA
*Hs*TRAF6_R125A_Reverse	AATCTCAGCTTTTGCAAAATTGTCTGGAAATAGTTGATTTTC
*Hs*TRAF6_S129A_Forward	GAGATTCTTGCGCTGATGGTGAAATGTCCAAATGAAGGTTGT
*Hs*TRAF6_S129A_Reverse	CACCATCAGCGCAAGAATCTCACGTTTTGCAAAATTGTCTGG
*Hs*TRAF6_E144A_Forward	CACAAGATGGCGCTGAGACATCTTGAGGATCATCAAGCACAT
*Hs*TRAF6_E144A_Reverse	ATGTCTCAGCGCCATCTTGTGCAAACAACCTTCATTTGGACA
*Hs*TRAF6_R146A_Forward	ATGGAACTGGCGCATCTTGAGGATCATCAAGCACATTGTGAG
*Hs*TRAF6_R146A_Reverse	CTCAAGATGCGCCAGTTCCATCTTGTGCAAACAACCTTCATT
*Hs*TRAF6_I73D_L75R_Forward	CGACTGCCGGATGGCATTACGAGAAGCAGTGCAAACGCCATGC
*Hs*TRAF6_I73D_L75R_Reverse	CATCCGGCAGTCGGGGCATTCATACTTGCTTTCCAGGGGTGGGTC
*Hs*TRAF6_R88A_Forward	GGCCATGCGTTCTGCAAAGCCTGCATCATAAAATCAATAAGG
*Hs*TRAF6_R88A_Reverse	GAACGCATGGCCGCATGGCGTTTGCACTGCTTCTCG
*Hs*TRAF6_F118A_Forward	CTAGCTCCAGACAATTTTGCAAAACGTGAGATTCTTTCTCTGATG
*Hs*TRAF6_F118A_Reverse	GTCTGGAGCTAGTTGATTTTCCAGCAGTATTTCATTGTCAACTGG
*Dr*TRAF6_RZ1_Reverse	AGTCCTCGAGGGCAAACCGGCACTGAGA
Ubc13_C87K_Forward	GGAAGAATAAAATTAGATATTTTGAAAGATAAGTGG
Ubc13_C87K_Reverse	AATATCTAATTTTATTCTTCCCAACTTGTCTACATT
Ubc13_K92T_K94Q_Forward	ATTTTGACTGATCAGTGGTCCCCAGCACTGCAGATCCGC
Ubc13_K92T_K94Q_Reverse	CTGATCAGTCAAAATATCTAATTTTATTCTTCCCAACTTGTCTACATT

Supplementary Table 2. Ordered gene.

*Dr*TRAF6_RZ3 (gBlock, Integrated DNA Technologies)	GCGCATATGCCTACTGACCAGCAAGGCTATGATGTAGAGTTTGACCCTCCACTTGAAAGCAAGTATGAGTGCCCTATCTGTCTGATGGGTCTCCGCTCGGCAGTACAGACCCCATGTGGCCATCGCTTCTGCGACTCGTGCATCCGGAAATCCATCCGCGACACGGGGCAGAAATGTCCAGTTGACAACGAGGTGCTGCTTGAGGAACAACTTTTCCCTGATAACTTTGCCAAACGTGAGATCCTCTCACTCACCGTCAAGTGTTCTAACTTTGGATGCAGTGAAAAAATGGAGTTGCGCCAATTAGAGAAACACTTGTCTCAGTGCCGGTTTGCCACTGCGCCGTGCCCTCAATGTCAGGAGTCTGTTCCGATGAGCCACCTGGATGAACATAAGAGCCAGCATTGCTTACAGCGGATTATGACCTGCCCTGACTGTGCTGGGAGCTTTGTGTATGCTGTCAAACAGAGTCATGAACAGTTTTGTCCTTTCGCCAATCTCGAGCGC

Supplementary References

1. Eddins, M. J., Carlile, C. M., Gomez, K. M., Pickart, C. M. & Wolberger, C. Mms2-Ubc13 covalently bound to ubiquitin reveals the structural basis of linkage-specific polyubiquitin chain formation. *Nat Struct Mol Biol* **13**, 915-920, doi:10.1038/nsmb1148 (2006)
2. Yin, Q. *et al.* E2 interaction and dimerization in the crystal structure of TRAF6. *Nat Struct Mol Biol* **16**, 658-666, doi:10.1038/nsmb.1605 (2009).
